# Urothelial carcinoma of the sarcomatoid variant in a young patient with spina bifida: a case report and review of the literature

**DOI:** 10.1186/1757-1626-2-9381

**Published:** 2009-12-22

**Authors:** Michael Nomikos, Prodromos Philippou, Chrysa Glava, Dimitrios Delakas

**Affiliations:** 1 Department of Urology, "Asklipieion" General Hospital, Athens, Greece; 2 Department of Pathology, University of Athens, Greece

## Abstract

**Introduction:**

Patients with neurogenic bladder due to spina bifida are considered to be at increased risk for aggressive bladder cancer. We present a unique case of a 32-year-old woman with spina bifida diagnosed with sarcomatoid urothelial carcinoma of the bladder and report diagnosis and management.

**Case presentation:**

A 32 year old woman with neurogenic bladder managed with intermittent self catheterisations, presented with gross hematuria. On cystoscopy, she had a bulky bladder mass on the posterior bladder wall. Bladder biopsies revealed sarcomatoid variant of bladder transitional cell carcinoma. Treatment included radical cystectomy with ileal conduit diversion and adjuvant chemotherapy with excellent intermediate term follow up.

**Conclusion:**

Patents with neurogenic bladder managed with intermittent self catheterisations need periodical follow up due to increased risk for aggressive bladder cancer. Immediate radical cystectomy with adjuvant chemotherapy is the suggested treatment approach.

## Introduction

Patients with neurogenic bladder due to spina bifida (SB) are considered to be at increased risk for bladder cancer. These tumours are composed of urothelial, glandular or small cell component showing variable degrees of differentiation, while a small subset may have a prominent myxoid stroma [1]. We report a case of bladder cancer of the sarcomatoid variant in a young woman with neurogenic bladder due to SB, and we present the diagnostic evaluation, management and intermediate-term outcome.

## Case presentation

**Figure 1:**
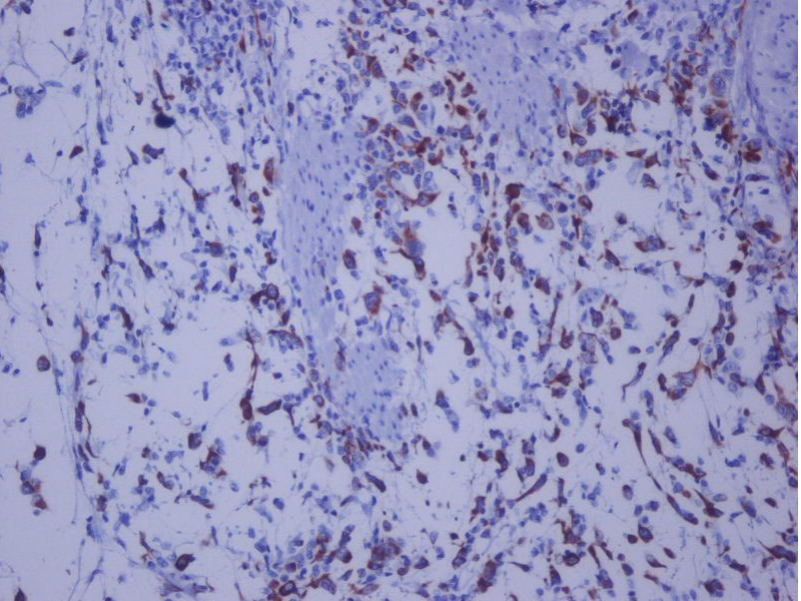
**Hematoxylin-eosin × 400 showing high frade urothelial carcinoma of the bladder**.

**Figure 2:**
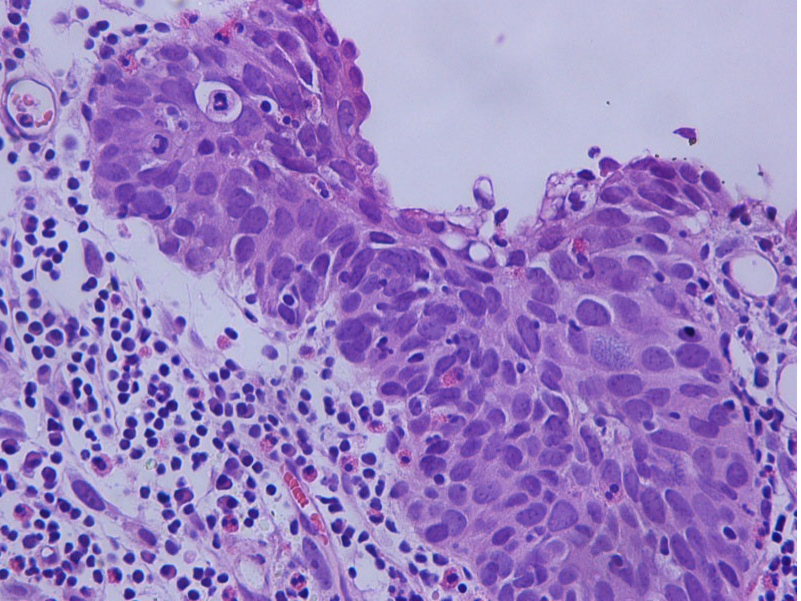
**Cytokeratin 18 immunostaining ×200: positive for malignant spindle cell tumor in the urinary bladder**.

A 32-year-old Greek white woman with neurogenic bladder due to spina bifida managed with intermittent self catheterizations since puberty; presented with gross hematuria and recurrent urinary tract infections. There was no history of bladder cancer in patient's family. Cystoscopy showed a bulky solid mass occupying the posterior bladder wall. Computed tomography didn't show nodal or distant metastases. Bladder biopsies revealed muscle-invasive sarcomatoid urothelial carcinoma with carcinoma in situ of the bladder neck. Radical cystectomy with anterior pelvic exenteration and ileal conduit formation was performed. Microscopically, the tumor was a high-grade urothelial carcinoma, Grade III according to the classification of the World Health Organization (WHO) of 1973, invading the whole bladder-wall and the pericystic lipoid tissue (stage pT3a). A large subset of the tumor assumed a spindle cell/sarcomatoid appearance with high mitotic rate and atypical mitoses. Immunohistochemically, the tumor cells were positive for the epithelial membrane antigen (EMA), cytokeratin 18 (CK18) and Vimentin. Some tumor cells were positive for CD117 (c-kit), whereas all of them were negative for CK7, CK20, Desmin and CD34 (Figure 1, and Figure 2). These histologic and immunohistochemical evidence of both epithelial and mesenchymal differentiation classify this tumor, as sarcomatoid variant of urothelial carcinoma without heterologous elements. The patient has completed 4 cycles of gemcitabine and carboplatine and at 9 months of follow-up remains disease free

## Discussion

Patients with neurogenic bladder are considered to be at high risk for bladder malignancies, with the vast majority of data coming from series of spinal cord injured patients [2]. Less than 20 cases of patients with spina bifida (SB) and urothelial bladder cancer have been reported in the literature, mainly presenting with locally advanced or metastatic disease [3]. Patients present with nonspecific symptoms, since gross hematuria was the main symptom in only 63% of cases. The median survival was only 6 months [4].

This is the first report of bladder urothelial carcinoma of the sarcomatoid variant diagnosed in a young patient with spina bifida.

Recent molecular studies support a monoclonal cell origin and suggest that clonal divergence may occur during tumor progression and differentiation [5].

Sarcomatoid carcinoma is a biphasic malignant neoplasm exhibiting morphologic and/or immunohistochemical evidence of epithelial and mesenchymal differentiation By immunohistochemistry, epithelial elements react with cytokeratins, whereas stromal elements react with Vimentin or specific markers corresponding to the mesenchymal differentiation. Microscopically, it is composed of spindle cell/sarcomatoid elements with high mitotic rate and atypical mitoses [6].

In conclusion, high index of clinical suspicion and appropriate usage of immunohistochemical techniques are essential for fast diagnosis of this rare clinical entity. Primary radical cystectomy and adjuvant chemotherapy may improve outcomes.

## Consent

Written informed consent was obtained from the patient for publication of this case report and accompanying images. A copy of the written consent is available for review by the Editor-in-Chief of this journal.

## Competing interests

The authors declare that they have no competing interests.

## Authors' contributions

MN operated on the patient, analysed and interpreted the data and revised the manuscript PF was a major contributor to the writing of the case report and completed the literature search CG performed the histological examination of the surgical specimen (urinary bladder with uterus) DD contributed in writing and revision of the manuscript. All authors read and approved the final manuscript.
